# HIV Infection and Awareness among Men Who Have Sex with Men–20 Cities, United States, 2008 and 2011

**DOI:** 10.1371/journal.pone.0076878

**Published:** 2013-10-23

**Authors:** Cyprian Wejnert, Binh Le, Charles E. Rose, Alexandra M. Oster, Amanda J. Smith, Julia Zhu, Gabriela Paz-Bailey for the NHBS Study Group

**Affiliations:** Division of HIV/AIDS Prevention, National Center for HIV, Viral Hepatitis, STD and TB Prevention, Centers for Disease Control and Prevention, Atlanta, Georgia, United States of America; Rollins School of Public Health, Emory University, United States of America

## Abstract

Over half of HIV infections in the United States occur among men who have sex with men (MSM). Awareness of infection is a necessary precursor to antiretroviral treatment and risk reduction among HIV-infected persons. We report data on prevalence and awareness of HIV infection among MSM in 2008 and 2011, using data from 20 cities participating in the 2008 and 2011 National HIV Behavioral Surveillance System (NHBS) among MSM. Venue-based, time-space sampling was used to recruit men for interview and HIV testing. We analyzed data for men who reported ≥1 male sex partner in the past 12 months. Participants who tested positive were considered to be aware of their infection if they reported a prior positive HIV test. We used multivariable analysis to examine differences between results from 2011 vs. 2008. HIV prevalence was 19% in 2008 and 18% in 2011 (p = 0.14). In both years, HIV prevalence was highest among older age groups, blacks, and men with lower education and income. In multivariable analysis, HIV prevalence did not change significantly from 2008 to 2011 overall (p = 0.51) or in any age or racial/ethnic category (p>0.15 in each category). Among those testing positive, a greater proportion was aware of their infection in 2011 (66%) than in 2008 (56%) (p<0.001). In both years, HIV awareness was higher for older age groups, whites, and men with higher education and income. In multivariable analysis, HIV awareness increased from 2008 to 2011 overall (p<0.001) and for all age and racial/ethnic categories (p<0.01 in each category). In both years, black MSM had the highest HIV prevalence and the lowest awareness among racial/ethnic groups. These findings suggest that HIV-positive MSM are increasingly aware of their infections.

## Introduction

Men who have sex with men (MSM) are at increased risk of HIV infection. MSM are the only group for which the annual number of diagnoses of HIV infection increased from 2007 to 2010 from 28,077 to 30,573 [1]. Male-to-male sexual contact accounted for 65% of all HIV diagnoses reported in 2011 [1]. Further, CDC estimates that the number of incident HIV infections (new HIV infections, whether diagnosed or not) increased 12% among MSM from 2008 to 2010 [2]. Of the 47,500 estimated incident HIV infections in 2010 in the United States, 63% were estimated to have occurred among MSM [2]. Among MSM, blacks are disproportionately affected by HIV. Black MSM accounted for 42% of all estimated incident HIV infections attributed to male-to-male sexual contact in 2010 [2].

Efforts to reduce the number of new HIV infections among MSM currently include efforts to increase awareness of one’s HIV infection status. HIV-infected persons who are not aware of their HIV infection status are more likely to engage in behaviors that place their partners at risk of HIV transmission [3]; moreover, estimates indicate that this group accounts for the majority of sexual HIV transmissions in the United States [4]. For this reason, increasing the proportion of HIV-infected persons who are diagnosed, and are therefore aware of their HIV serostatus, is one of the key objectives of the U.S. National HIV/AIDS Strategy, released in July 2010 [5].

CDC’s National HIV Behavioral Surveillance System (NHBS) was initiated in 2003 to monitor HIV-associated behaviors by conducting surveys in populations at high risk of HIV infection, including MSM [6]. During the first round of NHBS among MSM (2003–2005), HIV testing was piloted in five of 15 participating cities [7]; HIV testing was incorporated as a routine part of NHBS operations in all 21 cities participating in 2008 [8] and in all 20 cities that participated in NHBS in 2011. By conducting HIV testing, NHBS provides data to monitor HIV prevalence overall and among various subgroups and to determine how prevalence changes over time. By matching HIV test results with survey data, it is also possible to calculate what proportion of HIV-positive participants are aware of their HIV infection and how this changes over time in similar populations. We used NHBS data to determine HIV prevalence and awareness of infection among MSM interviewed in 20 cities and compare HIV prevalence and awareness among MSM interviewed in 2008 and 2011.

## Methods

NHBS staff members working in 20 metropolitan statistical areas (MSAs) with the largest burden of acquired immune deficiency syndrome (AIDS) collected cross-sectional behavioral data and conducted HIV testing among MSM in 2008 and 2011 using venue-based, time-space sampling (VBS). The 20 MSAs (referred to as “cities” hereafter) sampled in both 2008 and 2011 were: Atlanta–Sandy Springs–Marietta, GA; Baltimore–Towson, MD; Boston–Cambridge–Quincy, MA–NH: Boston–Quincy Division; Chicago–Joliet–Napierville, IL: Chicago–Joliet–Napierville Division; Dallas–Fort Worth–Arlington, TX: Dallas–Plano–Irving Division; Denver–Aurora–Broomfield, CO; Detroit–Warren–Livonia, MI: Detroit–Livonia–Dearborn Division; Houston–Sugar Land–Baytown, TX; Los Angeles–Long Beach–Santa Ana, CA: Los Angeles–Long Beach–Glendale Division; Miami–Ft. Lauderdale–Pompano Beach, FL: Miami Division; New Orleans–Metairie–Kenner, LA; New York–Northern New Jersey–Long Island, NY–NJ–PA: New York–White Plains–Wayne Division; New York–Northern New Jersey–Long Island, NY–NJ–PA: Nassau–Suffolk Division; New York–Northern New Jersey–Long Island, NY–NJ–PA: Newark–Union Division; Philadelphia–Camden–Wilmington, PA–NJ–DE–MD: Philadelphia Division; San Diego–Carlsbad–San Marcos, CA; San Francisco–Oakland–Fremont, CA: San Francisco–San Mateo–Redwood City Division; San Juan–Caguas–Guaynabo, PR; Seattle–Tacoma–Bellevue, WA: Seattle–Bellevue–Everett Division; and Washington–Arlington–Alexandria, DC–VA–MD–WV: Washington–Arlington–Alexandria Division.

NHBS VBS procedures have been previously published [9], [10] and are briefly summarized here. First, NHBS staff identified appropriate venues (e.g., bars, social organizations, sex venues) and days and times when men frequented those venues. In 2008, only venues in which 75% of men attending were MSM were eligible for inclusion; in 2011, because venues had become more integrated by sexual orientation, this threshold was lowered to 50%. Second, venues and corresponding day/time periods were chosen randomly each month for recruitment events. Third, staff members systematically approached men to screen for eligibility at each recruitment event. Men eligible to be interviewed were aged ≥18 years, residents of a participating city, able to complete the interview in English or Spanish, and willing and able to provide informed consent. Additionally, in 2011, only men who reported having ever had oral or anal sex with another man were interviewed. Although eligibility criteria differed, the same analysis criteria were used for both years in this analysis. After participants provided informed consent, trained interviewers conducted anonymous face-to-face interviews using a standardized questionnaire about demographics, HIV-associated behaviors and use of prevention and testing services. Participants who completed the interview were given the option complete an HIV test. HIV testing was performed by collecting blood or oral specimens for either rapid testing in the field or laboratory-based testing followed by laboratory confirmation. Participants received incentives for participating in the interview and the HIV test. The incentive format (cash or gift card) and amount varied by city based on formative assessment and local policy. A typical incentive included $25 for completing the interview and $25 for providing a specimen for HIV testing. Although incentives varied across cities, each of the 20 cities used the same incentive amounts in 2008 and 2011.

### Ethics Statement

Activities for NHBS were approved by local institutional review boards (IRB) for each of the 20 participating cities. NHBS activities were determined to be research in which the Centers for Disease Control and Prevention were not directly engaged and, therefore, did not require review by CDC IRB [11], [12]. All participants were explicitly assured during the recruitment process of the anonymous nature of the survey and the HIV testing. No personal identifiers were collected during enrollment, interview, or testing. All participants provided verbal informed consent to take part in the interview and to be tested for HIV. Verbal consent was documented electronically on the survey instrument by interviewers for all participants and on hard copy as required by local IRBs. Because data collection was anonymous, written consent was not possible and participant names or other personal identifiers were not linked to any NHBS instruments. All consent procedures, including verbal consent, were approved by local IRBs (see supplemental material, Text S1, for specific IRB information).

### Measures

The two main outcomes in this analysis are HIV prevalence and awareness of infection. During the interview, participants were asked questions to determine if they had previously tested positive for HIV. After the interview, all participants were offered HIV testing. A nonreactive rapid test was considered a definitive negative result; a reactive (preliminary positive) rapid test result was considered a definitive positive only when confirmed by supplemental laboratory testing (e.g., Western blot, immunofluorescence assay, or nucleic acid amplification test) [13]–[15]. Participants with a confirmed positive HIV test result who reported having previously tested positive for HIV were considered to be aware of their infection. Demographic variables (age, race/ethnicity, education, and income) were analyzed as categorical variables.

### Analysis

Participants were excluded from this analysis if they did not report having had sex with another man during the previous 12 months; did not have a definitive positive or negative NHBS HIV test result; reported being HIV-positive, but had a negative NHBS HIV test result at the time of interview; did not complete the interview; or were determined by the interviewer to have provided responses with questionable validity. Unadjusted prevalence ratios (PRs) were calculated to explore differences across time and racial categories. Univariate analysis of racial disparities in prevalence and awareness of HIV-positive status was conducted separately for 2008 and 2011 data and using combined 2008 and 2011 data with similar results (results not shown). To further assess potential changes in HIV prevalence and awareness over time, we used a Poisson model with a robust standard error [16] calculated using PROC GENMOD in SAS v.9.3.2. Two models were used, one with HIV prevalence as the outcome and another with HIV awareness as the outcome. Each initial model included demographic variables (age, race/ethnicity, education, and income) as well as venue type (bars/clubs, sex venues, other venues), city, sexual identity, outness (‘have you told anyone you are attracted to men?’ Yes/No), and the interactions of these variables with interview year as fixed effects. Interaction terms of each variable with interview year were used to calculate adjusted prevalence ratios (aPR) comparing the outcome in 2011 to 2008 for each independent variable stratum. The interaction terms of venue type, outness, and sexual identity with interview year were evaluated for both models using a score test. Interaction terms for venue type with year were significant and included in both models of HIV prevalence and awareness. Outness and sexual identity were retained in the final models as controls; however interaction terms for outness and sexual identity with year did not contribute significantly to either model and were excluded. Because the goal of this analysis was to describe HIV prevalence and awareness and how these indicators may have changed across time among demographic subsets of the MSM population, behavioral and other risk factors were not considered in this analysis. VBS sampling weights were not available for these data (VBS sampling weights are currently under development for future NHBS data collection cycles). Venue type and city were included in each model to account for some of the methodological complexities associated with VBS.

## Results

Table 1 shows response rates for various stages of recruitment in 2008 and 2011. In the 20 cities included in this analysis, a total of 11,988 and 12,123 men were screened for participation in NHBS in 2008 and 2011, respectively. In 2008, 86% of men screened were eligible to complete the interview; however of men who were screened in 2008, 79% met 2011 eligibility criteria, meaning that they both met 2008 eligibility criteria and reported ever having sex with another during the interview. In 2011, 82% of men screened met the same 2011 eligibility criteria. Of those who completed the interview, 91% (in 2008) and 94% (in 2011) consented to HIV testing. Of those who consented to HIV testing, 84% (in 2008) and 91% (in 2011) met inclusion criteria for this analysis. The majority of men excluded from this analysis either did not consent to HIV testing (889 in 2008; 588 in 2011) or did not report having sex with another man in the past 12 months (1,173 in 2008, 514 in 2011); reasons are not mutually exclusive. Characteristics of the full analysis sample are presented in Table 2. A total of 7,847 MSM interviewed and tested in 2008 were included in analysis of HIV prevalence (Table 3), of which 1,520 had a confirmed HIV positive result and were included in analysis of awareness (Table 4). Similarly, 8,423 men interviewed and tested in 2011 were included in our analysis of HIV prevalence (Table 3). Of those, 1,556 had a confirmed HIV-positive test result during the NHBS interview and were included in our analysis of HIV awareness (Table 4).

**Table 1:** Response rates of men who have sex with men– National HIV Behavioral Surveillance System, 20 Cities, United States, 2008 and 2011.

	2008		2011	
	No.	(%)	No.	(%)
Approached	27,257		39,792	
Screened, of approached	11,988	(44)	12,123	(30)
Met 2008 eligibility^a^ and consented to interview, of screened	10,282	(86)	–	–
Met 2008 & 2011 eligibility^b^ and consented to interview, of screened	9,518	(79)	9,881	(82)
Completed NHBS interview, of eligible during interview year and consented to interview	10,233	(100)	9,828	(99)
Consented to HIV testing, of completed interview	9,344	(91)	9,240	(94)
Met analysis inclusion criteria^c^, of consented to testing	7,847	(84)	8,423	(91)

a All men were eligible for participation in NHBS in 2008.

b Only men who reported ever having sex with another man were eligible for interview in NHBS in 2011.

c Limited to men who reported sex with a man in the past 12 months, had a valid HIV test result, and provided valid interview data.

**Table 2:** Characteristics of men who have sex with men– National HIV Behavioral Surveillance System, 20 Cities, United States, 2008 and 2011.

	2008		2011	
	No.	(%)	No.	(%)
**Age (yrs)**				
18–24	1,834	(23)	2,182	(26)
25–29	1,470	(19)	1,626	(19)
30–39	2,163	(28)	1,978	(23)
≥40	2,380	(30)	2,637	(31)
**Race/Ethnicity**				
Hispanic/Latino	2,035	(26)	2,227	(26)
Black	1,849	(24)	2,216	(26)
White	3,355	(43)	3,338	(40)
Other^a^	603	(8)	622	(7)
**Education**				
Less than high school graduate	518	(7)	479	(6)
High school diploma or equivalent	1,851	(24)	2,056	(24)
Some college or technical college	2,585	(33)	2,876	(34)
College degree or higher education	2,893	(37)	3,011	(36)
**Annual Household Income**				
0 to $19,999	2,370	(30)	2,688	(32)
$20,000 to $39,999	2,003	(26)	2,084	(25)
$40,000 to $74,999	1,901	(24)	1,966	(23)
$75,000 or more	1,467	(19)	1,553	(18)
**Outness** b				
Yes	7,315	(93)	7,856	(93)
**Sexual Identity**				
Homosexual	6,308	(80)	6,874	(82)
Bisexual	1,434	(18)	1,427	(17)
Heterosexual	95	(1)	97	(1)
**Venue Type** c				
Bars/Clubs	5,156	(66)	5,516	(65)
Sex Venues	512	(7)	569	(7)
Other Venues^d^	2,179	(28)	2,338	(28)
**City**				
Atlanta, Georgia	343	(4)	502	(6)
Baltimore, Maryland	447	(6)	403	(5)
Boston, Massachusetts	198	(3)	345	(4)
Chicago, Illinois	516	(7)	440	(5)
Dallas, Texas	461	(6)	414	(5)
Denver, Colorado	449	(6)	486	(6)
Detroit, Michigan	312	(4)	412	(5)
Houston, Texas	436	(6)	494	(6)
Los Angeles, California	478	(6)	495	(6)
Miami, Florida	526	(7)	499	(6)
Nassau-Suffolk, New York	242	(3)	317	(4)
New Orleans, Louisiana	354	(5)	412	(5)
New York, New York	462	(6)	483	(6)
Newark, New Jersey	80	(1)	189	(2)
Philadelphia, Pennsylvania	440	(6)	514	(6)
San Diego, California	490	(6)	429	(5)
San Francisco, California	474	(6)	431	(5)
San Juan, Puerto Rico	313	(4)	335	(4)
Seattle, Washington	352	(4)	351	(4)
Washington DC	474	(6)	472	(6)
**Total**	**7,847**		**8,423**	

a Includes: American Indian or Alaska Native, Asian, Native Hawaiian or Pacific Islander, other race, or multiple races.

b Participants were asked if they had ever told anyone they were attracted to other men (Yes or No).

c Venue type refers to the type of venue the participant was recruited from.

d Other venues include gyms, restaurants, parks, street locations, social organizations, and other places where MSM congregate.

**Table 3:** HIV prevalence among men who have sex with men– National HIV Behavioral Surveillance System, 20 Cities, U.S., 2008 and 2011.

	2008			2011								
	Total	HIVPositive	(%)	Total	HIVPositive	(%)	PrevalenceRatio	95% CI^a^	p-value	aPR^b^	95% CI^a^	p-value
**Age (yrs)**												
18–24	1,834	194	(11)	2,182	252	(12)	1.09	0.92–1.30	0.33	1.06	0.85–1.31	0.62
25–29	1,470	215	(15)	1,626	244	(15)	1.03	0.87–1.22	0.77	0.95	0.78–1.16	0.63
30–39	2,163	459	(21)	1,978	381	(19)	0.91	0.80–1.02	0.12	0.9	0.76–1.05	0.18
≥40	2,380	652	(27)	2,637	679	(26)	0.94	0.86–1.03	0.19	0.93	0.8–1.07	0.30
**Race/Ethnicity**												
Black	1,849	531	(29)	2,216	665	(30)	1.04	0.95–1.15	0.37	1.09	0.94–1.27	0.25
Hispanic/Latino	2,035	356	(17)	2,227	342	(15)	0.88	0.77–1.01	0.06	1	0.83–1.2	1.00
White	3,355	530	(16)	3,338	459	(14)	0.87	0.78–0.98	0.019	0.93	0.79–1.09	0.37
Other^c^	603	103	(17)	622	84	(14)	0.79	0.61–1.03	0.08	0.82	0.62–1.08	0.17
**Education**												
Less than high school graduate	518	131	(25)	479	113	(24)	0.88	0.48–1.59	0.67	0.89	0.69–1.15	0.36
High school diploma or equivalent	1,851	434	(23)	2,056	453	(22)	0.94	0.75–1.19	0.63	0.94	0.8–1.11	0.48
Some college or technical college	2,585	547	(21)	2,876	587	(20)	0.94	0.84–1.05	0.29	0.98	0.84–1.14	0.78
College degree or higher education	2,893	408	(14)	3,011	402	(13)	0.96	0.87–1.07	0.49	1.02	0.87–1.2	0.81
**Annual Household Income**												
0 to $19,999	2,370	625	(26)	2,688	660	(25)	0.93	0.85–1.02	0.14	1.01	0.87–1.16	0.93
$20,000 to $39,999	2,003	380	(19)	2,084	417	(20)	1.05	0.93–1.19	0.40	1.06	0.9–1.25	0.46
$40,000 to $74,999	1,901	291	(15)	1,966	296	(15)	0.98	0.85–1.14	0.83	1.01	0.84–1.22	0.90
$75,000 or more	1,467	207	(14)	1,553	158	(10)	0.72	0.59–0.88	0.001	0.77	0.61–0.98	0.03
**City**												
Atlanta, Georgia	343	22	(6)	502	133	(26)	4.13	2.69–6.35	<.0001	4.95	3.15–7.76	<0.0001
Baltimore, Maryland	447	169	(38)	403	172	(43)	1.13	0.96–1.33	0.15	0.91	0.73–1.14	0.42
Boston, Massachusetts	198	24	(12)	345	35	(10)	0.84	0.51–1.37	0.48	0.84	0.52–1.34	0.46
Chicago, Illinois	516	93	(18)	440	84	(19)	1.06	0.81–1.38	0.67	0.97	0.74–1.28	0.84
Dallas, Texas	461	119	(26)	414	105	(25)	0.98	0.78–1.23	0.88	0.93	0.72–1.18	0.54
Denver, Colorado	449	70	(16)	486	71	(15)	0.94	0.69–1.27	0.68	0.89	0.65–1.22	0.46
Detroit, Michigan	312	44	(14)	412	71	(17)	1.22	0.86–1.73	0.26	1.11	0.77–1.6	0.57
Houston, Texas	436	113	(26)	494	84	(17)	0.66	0.51–0.84	0.001	0.72	0.54–0.95	0.02
Los Angeles, California	478	89	(19)	495	84	(17)	0.91	0.70–1.19	0.50	1.06	0.8–1.41	0.68
Miami, Florida	526	133	(25)	499	114	(23)	0.90	0.73–1.12	0.36	0.93	0.73–1.19	0.58
Nassau–Suffolk, New York	242	19	(8)	317	9	(3)	0.36	0.17–0.79	0.01	0.44	0.2–0.95	0.04
New Orleans, Louisiana	354	76	(21)	412	67	(16)	0.76	0.56–1.02	0.07	0.8	0.59–1.1	0.17
New York, New York	462	132	(29)	483	92	(19)	0.67	0.53–0.84	0.0007	0.65	0.51–0.83	0.0006
Newark, New Jersey	80	15	(19)	189	47	(25)	1.33	0.79–2.23	0.29	1.2	0.7–2.04	0.51
Philadelphia, Pennsylvania	440	48	(11)	514	62	(12)	1.11	0.78–1.58	0.58	0.81	0.56–1.18	0.27
San Diego, California	490	87	(18)	429	74	(17)	0.97	0.73–1.29	0.84	1.05	0.78–1.41	0.74
San Francisco, California	474	111	(23)	431	94	(22)	0.93	0.73–1.19	0.56	1.03	0.79–1.33	0.84
San Juan, Puerto Rico	313	36	(12)	335	30	(9)	0.78	0.49–1.23	0.29	0.66	0.4–1.1	0.11
Seattle, Washington	352	52	(15)	351	68	(19)	1.31	0.94–1.82	0.11	1.24	0.89–1.72	0.21
Washington DC	474	68	(14)	472	60	(13)	0.89	0.64–1.22	0.46	0.86	0.63–1.19	0.37
**Total**	**7,847**	**1,520**	**(19)**	**8,423**	**1,556**	**(18)**	**0.95**	**0.89–1.01**	**0.14**	**0.96**	**0.84–1.09**	**0.51**

a Confidence interval.

b Adjusted prevalence ratio; Model includes: all variables shown & their interactions with year, venue type & interaction with year, outness, & sexual identity as fixed effects. Referent = 2008.

c Includes MSM reporting American Indian or Alaska Native, Asian, Native Hawaiian or Pacific Islander, other race, or multiple races.

**Table 4:** HIV awareness^a^ among HIV-infected men who have sex with men– National HIV Behavioral Surveillance System, 20 Cities, U.S., 2008 and 2011.

	2008			2011								
	HIV Positive	Aware	(%)	HIV Positive	Aware	(%)	Prevalence Ratio	95% CI^b^	p-value	aPR^c^	95% CI	p-value
**Age (yrs)**												
18–24	194	61	(31)	252	123	(49)	1.55	1.22–1.98	0.0004	1.65	1.27–2.13	0.0002
25–29	215	91	(42)	244	139	(57)	1.35	1.11–1.63	0.0022	1.38	1.12–1.71	0.003
30–39	459	249	(54)	381	254	(67)	1.23	1.10–1.37	0.0002	1.41	1.21–1.65	<0.0001
≥40	652	453	(69)	679	516	(76)	1.09	1.02–1.17	0.0079	1.29	1.11–1.49	0.0007
**Race/Ethnicity**												
Black	531	219	(41)	665	357	(54)	1.30	1.15–1.47	<.0001	1.37	1.15–1.62	0.0004
Hispanic/Latino	356	194	(54)	342	216	(63)	1.16	1.02–1.31	0.020	1.33	1.12–1.58	0.001
White	530	394	(74)	459	394	(86)	1.15	1.08–1.23	<.0001	1.36	1.18–1.58	<0.0001
Other^d^	103	47	(46)	84	60	(71)	1.57	1.22–2.01	0.0005	1.67	1.29–2.17	<0.0001
**Education**												
Less than high school graduate	131	64	(49)	113	71	(63)	1.77	1.13–2.77	0.012	1.44	1.12–1.85	0.005
High school diploma or equivalent	434	204	(47)	453	258	(57)	1.21	0.93–1.56	0.15	1.42	1.21–1.68	<0.0001
Some college or technical college	547	322	(59)	587	390	(66)	1.21	1.07–1.38	0.0033	1.40	1.2–1.63	<0.0001
College degree or higher education	408	264	(65)	402	312	(78)	1.13	1.03–1.24	0.0089	1.45	1.24–1.69	<0.0001
**Annual Household Income**												
0 to $19,999	625	328	(52)	660	415	(63)	1.20	1.09–1.32	0.0002	1.45	1.25–1.68	<0.0001
$20,000 to $39,999	380	202	(53)	417	275	(66)	1.24	1.10–1.39	0.0003	1.53	1.29–1.81	<0.0001
$40,000 to $74,999	291	175	(60)	296	203	(69)	1.14	1.01–1.29	0.034	1.35	1.14–1.61	0.0007
$75,000 or more	207	144	(70)	158	128	(81)	1.16	1.04–1.31	0.011	1.38	1.15–1.66	0.0005
**City**												
Atlanta, Georgia	22	10	(45)	133	82	(62)	1.36	0.84–2.19	0.21	1.77	1.07–2.93	0.03
Baltimore, Maryland	169	45	(27)	172	54	(31)	1.18	0.84–1.65	0.33	1.37	0.96–1.94	0.08
Boston, Massachusetts	24	17	(71)	35	30	(86)	1.21	0.91–1.62	0.20	1.44	1.08–1.92	0.01
Chicago, Illinois	93	44	(47)	84	61	(73)	1.53	1.19–1.97	0.0008	1.72	1.33–2.24	<0.0001
Dallas, Texas	119	55	(46)	105	74	(70)	1.52	1.21–1.92	0.0003	1.83	1.42–2.37	<0.0001
Denver, Colorado	70	56	(80)	71	55	(77)	0.97	0.82–1.15	0.71	1.22	0.99–1.51	0.07
Detroit, Michigan	44	13	(30)	71	41	(58)	1.95	1.19–3.22	0.0083	2.26	1.36–3.76	0.002
Houston, Texas	113	87	(77)	84	57	(68)	0.88	0.74–1.05	0.17	1.25	1.01–1.56	0.04
Los Angeles, California	89	60	(67)	84	65	(77)	1.15	0.95–1.38	0.14	1.37	1.1–1.7	0.004
Miami, Florida	133	73	(55)	114	80	(70)	1.28	1.05–1.55	0.014	1.43	1.15–1.78	0.001
Nassau–Suffolk, New York	19	14	(74)	9	7	(78)	1.06	0.68–1.64	0.81	1.40	0.92–2.13	0.12
New Orleans, Louisiana	76	56	(74)	67	46	(69)	0.93	0.76–1.15	0.51	1.19	0.94–1.5	0.15
New York, New York	132	63	(48)	92	57	(62)	1.30	1.02–1.65	0.033	1.54	1.2–1.99	0.0008
Newark, New Jersey	15	11	(73)	47	31	(66)	0.90	0.62–1.30	0.57	0.83	0.54–1.28	0.41
Philadelphia, Pennsylvania	48	14	(29)	62	43	(69)	2.38	1.48–3.81	0.0003	2.67	1.65–4.32	<0.0001
San Diego, California	87	52	(60)	74	52	(70)	1.18	0.94–1.48	0.16	1.43	1.1–1.85	0.01
San Francisco, California	111	90	(81)	94	89	(95)	1.17	1.05–1.29	0.0028	1.34	1.13–1.6	0.001
San Juan, Puerto Rico	36	10	(28)	30	7	(23)	0.84	0.36–1.94	0.68	0.77	0.3–1.97	0.59
Seattle, Washington	52	44	(85)	68	55	(81)	0.96	0.81–1.13	0.59	1.12	0.91–1.38	0.28
Washington DC	68	40	(59)	60	46	(77)	1.30	1.02–1.66	0.033	1.72	1.31–2.25	<0.0001
**Total**	**1,520**	**854**	**(56)**	**1,556**	**1,032**	**(66)**	**1.18**	**1.12–1.25**	**<0.0001**	**1.43**	**1.24–1.64**	**<0.0001**

a HIV aware defined as MSM with a confirmed HIV-positive test result who reported a previous HIV-positive test result during NHBS interview.

b Confidence interval.

c Adjusted prevalence ratio; Model includes: all variables shown & their interactions with year, venue type & interaction with year, outness, & sexual identity as fixed effects. Referent = 2008.

d Includes MSM reporting American Indian or Alaska Native, Asian, Native Hawaiian or Pacific Islander, other race, or multiple races.

Minor differences in eligibility from 2008 to 2011 resulted in a larger sample of MSM included in this analysis from 2011 than in 2008, but did not alter the sample compositions. Table 2 shows demographic characteristics of MSM interviewed in 2008 and 2011. Both samples were diverse with respect to age, race/ethnicity, education, and income. In both 2008 and 2011, the largest groups were MSM aged 40 years or older (30% in 2008; 31% in 2011), whites (43% in 2008; 40% in 2011), those with a college degree or higher education (37% in 2008; 36% in 2011), and those with household income less than $20,000 annually (30% in 2008; 32% in 2011). In both years, the majority of MSM had told someone they were attracted to men (93% in both 2008 and 2011), identified as homosexual (80% in 2008; 82% in 2011), and were recruited in bars or clubs (66% in 2008; 65% in 2011). The demographic distributions of data collected in 2008 and 2011 are nearly identical. In most cases, the sample distributions vary by less than two percentage points. The largest differences (five percentage points or fewer) occurred for age: 28% of the 2008 sample was aged 30–39 while only 23% of the 2011 sample was aged 30–39; and race: 43% of the 2008 sample was white while only 40% of the 2011 sample was white. While large sample size resulted in statistically significant differences (p<0.05) for whites, blacks, and MSM aged 30–39, the absolute similarities in the distribution of demographic variables suggest the samples collected in 2008 and 2011 are comparable. Further, the multivariable models adjust for any differences across years for these variables.

**Figure 1:**
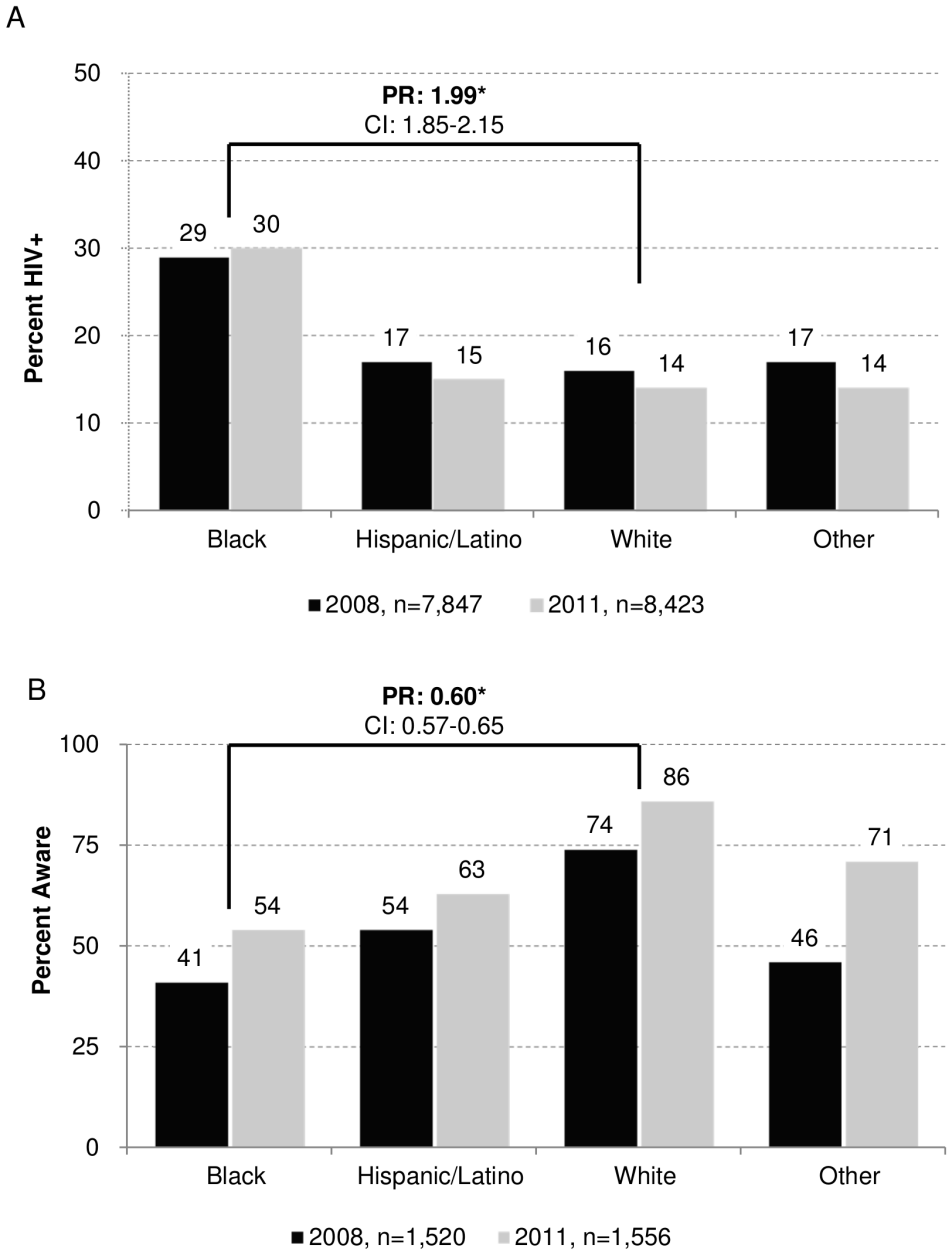
Prevalence and awareness of HIV status among MSM. (A) HIV prevalence of MSM interviewed in 2008 and 2011 by race/ethnicity. Compared to white MSM, black MSM were nearly twice (PR: 1.99) as likely to be HIV positive. (B) Awareness of HIV infection among MSM interviewed in 2008 and 2011 by race/ethnicity. Compared to white MSM, black MSM were 40% (PR: 0.60) less likely to be aware of their HIV infection status. *PRs (prevalence ratios) based on combined 2008, 2011 data. Separate analysis of 2008 and 2011 data produced similar results for prevalence and awareness in both 2008 and 2011 (not shown). CI: 95% confidence interval.

Table 3 shows HIV prevalence results for 2008 and 2011. Overall, 19% of MSM interviewed in 2008 and 18% of MSM interviewed in 2011 tested positive for HIV infection. In both years, HIV prevalence was higher in men of older age and lower in men with higher education and income. HIV prevalence was highest among black MSM (29% in 2008; 30% in 2011) and MSM interviewed in Baltimore (38% in 2008; 43% in 2011). Figure 1a shows black MSM were nearly twice as likely to be HIV infected as white MSM (combined 2008, 2011 data PR = 1.99, p<0.0001). Table 3 also shows results from a multivariable model that compared HIV prevalence in 2011 to that in 2008. Overall, HIV prevalence in 2011 was not statistically different from 2008 (p = 0.51). No significant differences in HIV prevalence were observed across time by age, race/ethnicity, or education in the multivariable model (p>0.15). However, there was a significant decrease in prevalence for MSM earning over $75,000 annually (14% in 2008; 10% in 2011; p = 0.03). Significant differences (p<0.05) in prevalence across time were observed in four cities: Atlanta, GA; Houston, TX; Nassau-Suffolk, NY; and New York City, NY.

Data on awareness of HIV status among 1,520 MSM from 2008 and 1,556 MSM from 2011 who tested positive for HIV infection are presented in Table 4. Overall, 56% of MSM who tested positive for HIV infection in 2008 and 66% of MSM who tested positive in 2011 reported being aware of their HIV infection at the time of interview. In 2008 and 2011, awareness of infection was higher in men of older age and higher education and income. White MSM had the highest percentage aware (74% in 2008; 86% in 2011), while black MSM had the lowest percentage aware (41% in 2008; 54% in 2011). In the combined data, black MSM were 40% less likely to be aware of their infection than white MSM (PR = 0.60, p<0.0001), as shown in Figure 1b. In addition to black MSM, the lowest percentages of HIV-positive men who were aware of their infection were observed among MSM interviewed in Baltimore (27% in 2008; 31% in 2011) and San Juan, Puerto Rico (28% in 2008; 23% in 2011). In the multivariable model, overall awareness of positive HIV status was significantly higher in 2011 than in 2008 (aPR = 1.43, p<0.0001). In the same model, awareness was significantly higher in 2011 than 2008 for all demographic subgroups considered in the model. In most cases, the difference is highly significant (p<0.001). The largest increases in awareness occurred among MSM aged 18–24 years (aPR = 1.65, p = 0.0002) and MSM of other race or multiple races (aPR = 1.67, p<0.0001). Eighteen of 20 cities experienced an increase in awareness, indicated by an aPR>1.0. Of those, the increase was statistically significant (p<0.05) in 13 cities.

## Discussion

HIV prevalence among MSM sampled in 20 U.S. cities remained stable from 2008 to 2011. Awareness of HIV infection among HIV-infected MSM increased overall and among all age, race/ethnicity, education, and income strata. Consistent with other reports [1], minority MSM, especially black MSM, experienced a disproportionate burden of HIV in both years. Further, in both years, black MSM were most likely to be HIV-infected and least likely to be aware of their infection. Minority MSM remain an important target for efforts that promote testing and reduce risk and stigma associated with HIV.

Several aspects of these results require further discussion. First, given that persons living with a diagnosis of HIV are living longer and that new infections continue to occur [1], [2], we might expect to see increasing, not stable, prevalence. However, the increase in prevalence we would expect based on increased persons living with a diagnosis of HIV is likely too small to detect in NHBS over a three-year time span. Purcell et al. [17] estimate there were between 4.3 and 5.4 million MSM living in the United States in 2008. The number of MSM living with a diagnosis of HIV increases by approximately 20,000 each year (new infections [2] minus deaths [1]), which would produce an absolute increase in prevalence of less than one-half of one percent of the MSM population over three years. If the size of the MSM population is increasing, as is the rest of the U.S. population [18], the expected increase would be further reduced. One important difference between the population of HIV-infected MSM and NHBS is the age distribution; NHBS captures a younger sample of MSM. In 2008, an estimated 80% of MSM living with HIV in the United States were 35 years or older. In NHBS, 56% of MSM included in this analysis were aged 40 years or older. Consequently, prevalence measured in NHBS may not be affected by the increased life expectancy of persons living with HIV, as NHBS captures a young sample of MSM and older HIV-positive MSM may be less likely to be captured by our sampling strategy.

Second, we observed significant increases from 2008 to 2011 in awareness, defined as the percentage of MSM with a positive HIV test result who reported having previously tested positive for HIV, in all demographic subgroups after accounting for possible differences in sample composition due to venue type, sexual identity, outness, and city. It is not possible to fully exclude a methodological change contributing to this finding. However, most aspects of the sampling methodology, including the questionnaire items assessing awareness and prevalence, were unchanged from 2008 to 2011. One major change in methods was the inclusion of sexual behavior questions during eligibility screening. Requiring disclosure of sexual behaviors at the time of screening could have differentially selected participants that were more comfortable with disclosing their sexuality. However, once participants agreed to be screened, response rates at each stage of interview were consistent from 2008 to 2011. While procedures for approaching and recruiting men to be screened did not change from 2008 to 2011, a lower percentage of men approached in 2011 agreed to be screened than in 2008. It is possible an altruism bias (such that more altruistic MSM were more likely to participate) favored men who were more likely to be aware of their infection. However, the two samples were consistent with respect to all other variables considered and it is unlikely that such an altruism bias would greatly affect awareness and no other variable in the analysis.

Our results suggest that HIV-positive venue-attending MSM living in cities with high AIDS burden are increasingly aware of their infection. Because only two time points are available, we are not able to determine whether this represents a trend; future NHBS data will allow for trend analysis to determine if awareness continues to increase among MSM. However, the demographic characteristics of participating men in 2008 and 2011 are nearly identical, suggesting comparable populations of MSM were sampled during each year.

Our finding that the percentage aware increased substantially from 2008 to 2011 can be interpreted in two ways. One interpretation is that a higher proportion of HIV-infected MSM had been diagnosed in 2011 than in 2008, meaning that fewer were unaware of their infection. Recent HIV case surveillance data show an increase in the number of HIV diagnoses attributable to MSM from 2008 to 2011 [1]. Our results show stable HIV prevalence but a substantial increase in awareness of infection, suggesting the increase in observed HIV diagnoses in case surveillance data may, in part, be due to a higher proportion of HIV infections being diagnosed rather than solely to an increase in the number of individuals infected with HIV. While the two analysis populations are not directly comparable, the relative increase in awareness presented here is consistent with the increase in persons living with a diagnosis of HIV as reported by case surveillance [1]. Such an interpretation suggests recent efforts to increase HIV testing among MSM, such as CDC’s Expanded Testing Initiative [19] which funded expanded HIV testing in jurisdictions covering 17 of the 20 NHBS cities (cities not covered: Denver, CO; San Juan, PR; and Seattle, WA) from 2007 to 2010, and other testing efforts [20], [21] might be having a favorable effect on HIV testing and awareness of infection. An analysis of NHBS-MSM data, similar to that presented here, found a significant increase in testing in the past 12 months among MSM from 2008 to 2011 [22].

Another possible explanation for increased awareness in our analysis is that, for some reason, MSM with diagnosed HIV infection were more willing to disclose their HIV status to interviewers in 2011 than in 2008. NHBS operational procedures and questions regarding HIV status did not change between 2008 and 2011. However, participants may have been more open about their status in 2011 if stigma associated with HIV infection or homosexuality is decreasing, for which some evidence exists [23], [24].

Further research is needed to determine definitively whether the observed increase in awareness is due to an increase in the proportion of HIV-positive MSM who are diagnosed and aware of their infection or an increase in the proportion of HIV-positive MSM who are willing to disclose their HIV infection status or some combination of these two factors. However, both outcomes can represent progress in the fight against HIV. Increasing the proportion of HIV-infected individuals who know their infection status is a critical step toward treatment and prevention of transmission, and a key benchmark of the U.S. National HIV/AIDS Strategy [5]. Similarly, an increase in the proportion of HIV-positive MSM willing to reveal their infection status suggests decreased stigma against HIV and may signal increased willingness among HIV-positive MSM to seek treatment for their infection or to be open about their infection with partners or others. Both internalized and external stigma are known to negatively impact the health of HIV-positive individuals through delayed medical care-seeking and decreased adherence to antiretroviral medication [25]. Further, HIV-related stigma negatively affects prevention efforts by promoting avoidance of HIV testing among uninfected and undiagnosed individuals and reducing the likelihood of disclosure of HIV status to others by diagnosed HIV-infected persons [25]–[30]. Finally, the two explanations are not mutually exclusive. Reduced stigma may increase testing, resulting in increased diagnoses or increased testing could result in greater acceptance and reduced stigma.

The analysis presented here is subject to several limitations. First, MSM were recruited in cities with high AIDS burden and results may not be generalizable to all cities or all MSM. Cities with high AIDS burden are often the focus of increased prevention and testing campaigns, initiatives, and resources [19], [31]. Further research is needed to determine whether the observed increase in awareness is occurring nationwide or is limited to areas with increased HIV prevention resources. Second, our measure of awareness is based on self-reported data and may be subject to reporting bias. Third, data are not weighted to account for the complex sampling methodology required to locate, recruit, and interview MSM. VBS sampling weights are currently under development for future NHBS cycles. While weighting is not available for data presented here, variables typically correlated with VBS sampling weights (race/ethnicity, venue type, age, and city) were included in the analysis model to adjust for sampling differences and clustering. One potential implication of unweighted analysis is that point estimates may be biased by over- or under-represented subgroups of the population. Researchers should use caution when interpreting point estimates, especially those for the total sample. Multivariate analysis of differences across years should not be affected by unweighted analysis, especially given the consistency of data distribution across years. Unweighted analysis may also underestimate standard error of the estimates [32]. However, the magnitude of the results reported here is such that the conclusions would not change even if standard error were substantially underestimated. A fourth limitation of this analysis is that the survey population is limited to MSM who attend venues; MSM who do not attend venues may, or may not, differ from the survey population on key outcomes. For example, older MSM (who are also more likely to be HIV-infected) may be less likely to attend venues than younger MSM. Fifth, the analysis presented here is limited to two time points and may not represent a trend. Sixth, it is not possible to completely rule out the possibility of a systematic or methodological bias in our results. However, as is stated throughout this text, most NHBS procedures remained unchanged from 2008 to 2011 and those that did change do not appear to have resulted in different samples. Consequently, a methodological bias is unlikely. Finally, the analysis presented here did not include risk behaviors. Analysis of associations between prevalence, awareness, risk behaviors, and how these interact across time is beyond the scope of this analysis.

## Conclusion

The analysis presented here suggests measurable progress is being made in the fight against HIV among MSM. We found stable HIV prevalence and significant increases in awareness of infection overall and in all demographic subgroups considered. The findings raise questions about whether ongoing efforts to increase the proportion of HIV-infected MSM who are aware of their status and decrease HIV related stigma might be having a favorable impact on MSM in cities with the highest burden of AIDS. Despite these gains, continued racial/ethnic disparities remain among MSM, especially for black MSM, who were most likely to be HIV infected and least likely to know about their infection.

## Supporting Information

Text S1 **List of MSM IRBs.**
(DOCX)

## References

[1] CDC (2013) Diagnoses of HIV Infection in the United States and Dependent Areas, 2011. HIV Surveillance Report.

[2] CDC (2013) Estimated HIV incidence in the United States, 2007–2010. HIV Surveillance Supplimental Report Atlanta, GA: Centers for Disease Control and Prevention.

[3] Marks G, Crepaz N, Senterfitt JW, Janssen RS (2005) Meta-analysis of high-risk sexual behavior in persons aware and unaware they are infected with HIV in the United States: implications for HIV prevention programs. J Acquir Immune Defic Syndr 39: 446–453. 16010168 10.1097/01.qai.0000151079.33935.79

[4] Marks G, Crepaz N, Janssen RS (2006) Estimating sexual transmission of HIV from persons aware and unaware that they are infected with the virus in the USA. Aids 20: 1447–1450. 16791020 10.1097/01.aids.0000233579.79714.8d

[5] The White House Office of National AIDS Policy (2010) National HIV/AIDS Strategy for the United States. Washington DC: The White House Office of National AIDS Policy.

[6] Gallagher KM, Sullivan PS, Lansky A, Onorato IM (2007) Behavioral surveillance among people at risk for HIV infection in the U.S.: the National HIV Behavioral Surveillance System. Public Health Reports 122 Suppl 132–38. 17354525 10.1177/00333549071220S106 PMC1804113

[7] CDC (2005) HIV prevalence, unrecognized infection, and HIV testing among men who have sex with men–five U.S. cities, June 2004-April 2005. MMWR Morb Mortal Wkly Rep 54: 597–601. 15973239

[8] CDC (2010) Prevalence and awareness of HIV infection among men who have sex with men –21 cities, United States, 2008. MMWR Morb Mortal Wkly Rep 59: 1201–1207. 20864920

[9] MacKellar DA, Gallagher KM, Finlayson T, Sanchez T, Lansky A, et al (2007) Surveillance of HIV risk and prevention behaviors of men who have sex with men–a national application of venue-based, time-space sampling. Public Health Reports 122 Suppl 139–47. 17354526 10.1177/00333549071220S107 PMC1804106

[10] Finlayson TJ, Le B, Smith A, Bowles K, Cribbin M, et al (2011) HIV risk, prevention, and testing behaviors among men who have sex with men–National HIV Behavioral Surveillance System, 21 U.S. cities, United States, 2008. MMWR Surveill Summ 60: 1–34. 22031280

[11] CDC (1999) Guidelines for defining public health research and public health non-research. Atlanta, GA: Centers for Disease Control and Prevention.

[12] Code of Federal Regulations (1991) Protection of human subjects. 45 CFR 46.

[13] CDC (2004) Notice to readers: Protocols for confirmation of reactive rapid HIV tests. MMWR Morb Mortal Wkly Rep 53: 221–222.

[14] CDC (1998) Update: HIV counseling and testing using rapid tests–United States, 1995. MMWR Morb Mortal Wkly Rep 47: 211–215. 9551881

[15] CDC (1989) Interpretation and use of the western blot assay for serodiagnosis of human immunodeficiency virus type 1 infections. MMWR Morb Mortal Wkly Rep 38: 1–7. 2501638

[16] Barros AJ, Hirakata VN (2003) Alternatives for logistic regression in cross-sectional studies: an empirical comparison of models that directly estimate the prevalence ratio. BMC Med Res Methodol 3: 21. 14567763 10.1186/1471-2288-3-21 PMC521200

[17] Purcell DW, Johnson CH, Lansky A, Prejean J, Stein R, et al (2012) Estimating the population size of men who have sex with men in the United States to obtain HIV and syphilis rates. Open AIDS J 6: 98–107. 23049658 10.2174/1874613601206010098 PMC3462414

[18] Machkum P, Wilson S (2011) Population distribution and change: 2000 to 2010. US Census Bureau. Washington DC.

[19] CDC (2011) Results of the Expanded HIV Testing Initiative–25 jurisdictions, United States, 2007–2010. MMWR Morb Mortal Wkly Rep 60: 805–810. 21697804

[20] CDC (2013) HIV testing trends in the United States, 2000–2011. Division of HIV/AIDS Prevention, National Center for HIV/AIDS, Viral Hepatitis, STD, and TB Prevention.

[21] CDC (2011) Highlights of CDC activities addressing HIV prevention among African-American gay, bisexual, and other men who have sex with men. In: Prevention CfDCa, editor.

[22] Cooley LA, Oster AM, Wejnert C, Le B, Rose CE, et al.. (2013) Increased HIV testing among men who have sex with men - National HIV Behavioral Surveillance System, 20 U.S. Metropolitan Statistical Areas, 2008 and 2011. Epidemic Intelligence Service Conference. Atlanta, GA. 10.1371/journal.pone.0104162 PMC4151966 25180514

[23] Herek GM, Capitanio JP, Widaman KF (2002) HIV-related stigma and knowledge in the United States: prevalence and trends, 1991–1999. Am J Public Health 92: 371–377. 11867313 10.2105/ajph.92.3.371 PMC1447082

[24] Glick SN, Golden MR (2010) Persistence of racial differences in attitudes toward homosexuality in the United States. J Acquir Immune Defic Syndr 55: 516–523. 20838226 10.1097/QAI.0b013e3181f275e0 PMC2974805

[25] Rintamaki LS, Davis TC, Skripkauskas S, Bennett CL, Wolf MS (2006) Social stigma concerns and HIV medication adherence. AIDS Patient Care STDS 20: 359–368. 16706710 10.1089/apc.2006.20.359

[26] Fortenberry JD, McFarlane M, Bleakley A, Bull S, Fishbein M, et al (2002) Relationships of stigma and shame to gonorrhea and HIV screening. Am J Public Health 92: 378–381. 11867314 10.2105/ajph.92.3.378 PMC1447083

[27] Emlet CA (2006) A comparison of HIV stigma and disclosure patterns between older and younger adults living with HIV/AIDS. AIDS Patient Care STDS 20: 350–358. 16706709 10.1089/apc.2006.20.350

[28] Babalola S (2007) Readiness for HIV testing among young people in northern Nigeria: the roles of social norm and perceived stigma. AIDS Behav 11: 759–769. 17191141 10.1007/s10461-006-9189-0

[29] Berendes S, Rimal RN (2011) Addressing the slow uptake of HIV testing in Malawi: the role of stigma, self-efficacy, and knowledge in the Malawi BRIDGE Project. J Assoc Nurses AIDS Care 22: 215–228. 21185751 10.1016/j.jana.2010.08.005

[30] Darrow WW, Montanea JE, Gladwin H (2009) AIDS-related stigma among Black and Hispanic young adults. AIDS Behav 13: 1178–1188. 19680800 10.1007/s10461-009-9601-7

[31] CDC (2013) Enhanced Comprehensive HIV Prevention Planning and Implementation for Metropolitan Statistical Areas Most Affected by HIV/AIDS.

[32] Karon JM, Wejnert C (2012) Statistical Methods for the Analysis of Time-Location Sampling Data. J Urban Health 89(3): 565–586. 22421885 10.1007/s11524-012-9676-8 PMC3368048

