# Supplementary material for: HIV Infection and Awareness among Men Who Have Sex with Men–20 Cities, United States, 2008 and 2011
Source: PLoS One. 2013 Oct 23;8(10):e76878. doi: 10.1371/journal.pone.0076878 (PMC3806782; doi:10.1371/journal.pone.0076878)
Supplement: Text S1 — List of MSM IRBs. (DOCX) [file pone.0076878.s001.docx]

| **City** | **Approving IRB for NHBS-MSM activities (2008 and 2011)** |
| --- | --- |
| ATLANTA | Georgia Department of Human Resources IRB (2008 only)  Emory University IRB (2008 only)  Georgia Department of Public Health (2011 only)  Georgia Department of Community Health (2011 only) |
| BALTIMORE | Maryland Department of Health and Mental Hygiene IRB  Johns Hopkins Bloomberg School of Public Health IRB |
| BOSTON | Massachusetts Department of Public Health Human Research Review Committee  Boston University Medical Center IRB (2011 only) |
| CHICAGO | Chicago Department of Public Health IRB |
| DALLAS | Texas Department of State Health Services IRB  Texas A&M University Office of Research Compliance |
| DENVER | Colorado Multiple IRB |
| DETROIT | Michigan Department of Community Health IRB |
| HOUSTON | Univ. of Texas Health Science Center Committee for the Protection of Human Subjects |
| LA | County of Los Angeles Public Health IRB |
| MIAMI | Florida Department of Health IRB  University of Miami IRB (2008 only) |
| NASSAU | State of New York Department of Health IRB |
| NEW ORLEANS | State of Louisiana Department of Health and Hospitals IRB  Louisiana State University Health and Sciences Center IRB |
| NYC | New York City Department of Health and Mental Hygiene IRB  National Development and Research Institute IRB (2008 only)  John Jay College of Criminal Justice IRB (2011 only) |
| NEWARK | State of New Jersey Department of Health and Senior Services IRB |
| PHILADELPHIA | City of Philadelphia Department of Public Health IRB |
| SAN FRANCISCO | Univ. of California, San Francisco Committee on Human Research |
| SAN JUAN | Univ. of Puerto Rico, Medical Sciences Campus IRB |
| SAN DIEGO | California Health and Human Services Agency Committee for the Protection of Human Subjects |
| SEATTLE | State of Washington Department of Social and Health Services IRB |
| DC | Government of the District of Columbia Department of Health IRB  George Washington University Medical Center IRB |

Activities for NHBS were approved by local institutional review boards (IRB) for each of the 20 participating cities. NHBS activities were determined to be research in which the Centers for Disease Control and Prevention were not directly engaged and, therefore, did not require review by CDC IRB. Below is a list of all reviewing IRBs by city. The IRBs listed reviewed local NHBS operations in the respective city.
